# Evolving Patient-Researcher Collaboration: An Illustrative Case Study of a Patient-Led Knowledge Translation Event

**DOI:** 10.2196/jopm.8756

**Published:** 2017-08-04

**Authors:** Jenny Leese, Sheila Kerr, Annette McKinnon, Erin Carruthers, Catherine Backman, Linda Li, Anne Townsend

**Affiliations:** ^1^ Arthritis Research Canada Richmond, BC Canada

**Keywords:** Patient engagement, research collaboration, knowledge translation, patient-led

## Abstract

Patient engagement occurs when patients actively collaborate in health research in ways that are meaningful to them. Resources to facilitate patient engagement have been developed, but their approach is mainly toward building competencies in the early stages of forming new practices of patient engagement. This paper describes a patient-led collaboration in rheumatology, in the context of an established patient-researcher partnership. Using a case study approach, we report on a research knowledge translation event, titled eROAR2013 (Reaching Out with Arthritis Research), led by members of the Arthritis Patient Advisory Board (APAB), which is a group of volunteer advocates living with arthritis based at Arthritis Research Canada. We provide an overview of APAB’s decade-long history, describe the planning and the event itself, and report on the challenges encountered, reflections and solutions pertinent for sustaining patient-researcher collaborative practices.

## Introduction

The motto “Nothing About Us Without Us” underpins patient engagement in health research [1]. Adopted by the global disability rights movement, it reflects the principle of participation and wider societal developments toward realizing citizen empowerment [2,3]. Similarly, the emergence of “patients as partners” is integral to patient-centred care and shared decision making [4,5,6]. These developments, underpinned by values and ethical concepts such as mutual respect, have laid a foundation for patient engagement in health research.

Patient engagement in research varies from minimal involvement to more participatory collaboration, and is broadly understood to occur when patients meaningfully and actively collaborate at any stage of the research process, from setting the research agenda to designing the research project, collecting data, and disseminating results [7,8,9,10]. Support for patient engagement continues to increase [1,7,11]. For example, research funding agencies in Canada, the United States (US), United Kingdom (UK) and elsewhere recommend patient engagement as a means to improve research relevance and quality [8,12,13]. Yet, despite the strong rationale for patient engagement in research [11], the process of patient-researcher collaboration is little understood [14,15]. The UK’s National Institute for Health Research national advisory group INVOLVE have provided guidance to researchers to plan public involvement in research [16]. Hewlett and colleagues have also suggested a framework for patient-research partners based on experiences of researchers and patients collaborating in rheumatology research in the UK. They describe practical aspects and identify challenges (eg, anxieties felt by patient partners taking on a new role) [15]. While these publications can guide efforts to begin cultivating patient engagement in research, examples of collaboration in established patient-researcher partnerships of engagement in research are scant.

In this paper we describe a patient-led collaboration in rheumatology, embedded in an established patient-researcher partnership of over 10 years. We report on a research knowledge translation event, titled eROAR2013 (Reaching Out with Arthritis Research), as an illustrative case study of patient engagement in research [17]. While the event illustrates patient engagement in the late stages of the research process, it builds on patient-researcher collaboration from study inception. eROAR2013 also presents an example of the dynamic process of research knowledge translation, which aims to reach stakeholders at all levels of the health system (eg, patients, the public, and health practitioners) to make research evidence for informing health decisions accessible [18].

The patient collaborators were members of the Arthritis Patient Advisory Board (APAB) [a], based at Arthritis Research Canada where the researcher collaborators are also based. We describe the role and development of APAB and report on the planning, preparation, and description of the event. Finally, we outline the challenges to emerge, report our reflections and suggest solutions in the collaborative process.

## The History of the Arthritis Patient Advisory Board (APAB)

APAB [a] is comprised of volunteer advocates with at least one form of arthritis who bring personal experience and arthritis knowledge to research decision making at Arthritis Research Canada [19]. APAB was created in 2001 as a patient representative body of Arthritis Research Canada (created in 2000) with a mission “to participate in all components and phases of arthritis research, and serve as a bridge between researchers, people with arthritis, and the community at large” [20]. Established with five members, APAB included 15 current members and nine alumni (19 women; 5 men) in 2013. [b] Alumni periodically provided knowledge, expertise and advice to support the current members, whose roles included, but were not limited to, identifying research topics, shaping the research design, participating in grant applications, co-authoring scientific papers, and attending conferences, as well as mentoring other APAB members, researchers, trainees, and research staff at Arthritis Research Canada.

**Figure 1:**
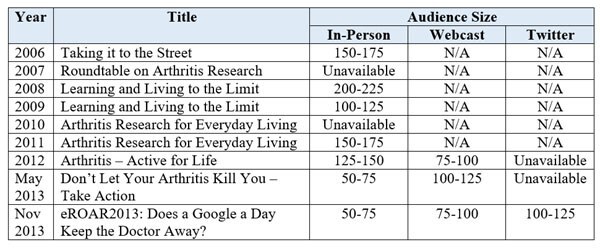
A History of ROAR’s reach

Since 2006, APAB members have organized annual knowledge translation events called Reaching Out with Arthritis Research (ROAR) in Vancouver, Canada, for people affected by arthritis. Each interactive event includes presentations from patients, researchers and health professionals providing practical information linking research to best practices in the prevention and management of arthritis in everyday life. This event also seeks to identify patients’ research interests by encouraging dialogue between patients and researchers, enabling opportunity for patients perspectives on research to be prioritized and incorporated in future patient-oriented research at Arthritis Research Canada. Participants are invited to an event via word of mouth, advertisements posted in local community centres and newspapers, as well as notices circulated online via social networking sites (eg, Facebook, Twitter), APAB’s quarterly newsletter, email distribution lists and newsletters of national organizations (eg, Arthritis Alliance of Canada, Arthritis Consumer Experts, and Canadian Arthritis Patient Alliance. Originally an in-person only event, since 2012, electronic media has extended the reach to a national and international audience of approximately 200 participants in total (Figure 1). [c]

In July 2013, APAB co-chair SK initiated a patient-researcher collaboration to raise awareness of preliminary findings from an ongoing research project titled “Exploring E-health Ethics & Multi-Morbidity” through a ROAR event [21,22]. Funded by a Canadian Institutes of Health Research (CIHR) catalyst grant competition (EPP-122907) in October 2012, the “Exploring E-health Ethics & Multi-Morbidity” project involved a transdisciplinary team with expertise in health services, rehabilitation, ethics and medical sociology, as well as health professionals, two patients and educators, led by AT and CB. APAB co-chair SK had acted as one of two patient collaborators on the project since its inception, providing perspectives that shaped the research topic and design.

## Planning and Preparation

Building on the well-established partnership with researchers at Arthritis Research Canada and drawing on their experience in hosting previous ROAR events, APAB collaborators were co-leaders in planning a ROAR event titled “eROAR2013: Does a Google a Day Keep the Doctor Away?” between August-November 2013. In September 2013, AT and JL were invited to attend one of APAB’s monthly meetings for its members (totalling 15 at the time). These structured yet informal monthly meetings routinely opened with a hot meal and “catching up,” which contributed to a culture of caring and respect for each other’s well-being, welcoming guests, and acknowledging individuals as members of the collective team. Following this, AT and JL described the research project “Exploring Ehealth Ethics & Multi-Morbidity” [21,22]. Of the total APAB membership (15 in 2013), approximately 10 were in attendance (either in-person or by phone) at the meeting with AT and JL to discuss how to ensure central concepts and messages from the research project would be accessible and meaningful to lay audiences. APAB’s co-chair SK played an invaluable lead role in enabling voices to be heard. APAB members combined their patient and collaborator roles and identities rather than compartmentalizing them, opening a participatory space for talk around living with arthritis and research tasks, bringing a richness and sensitivity to the discussions. Early in this planning process it was apparent that APAB’s culture and researchers’ interests aligned and fostered a participatory process underpinned by mutual respect for each other’s roles. By creating this informal, inclusive and interactive environment, decision making was a collaborative process from the beginning. Consistent with published frameworks, these elements illustrated a collaboration based on shared understanding and a recognition of multiple identities within the life contexts of APAB members [15].

SK and AT acted as representatives for APAB and the research team respectively. The aims and format of eROAR2013 were agreed upon and clearly laid out in the early stages of planning, which required dialogue and precise understanding between all parties (i.e. APAB members and the research team). Mutual agreement on strategies of communication for the event was also required. APAB collaborators led the preparation and dissemination of promotional materials for the event, including the level of language used, format and key distribution channels. There was an ongoing negotiation via emails between SK and AT with final promotional materials approved by APAB members. The range of speakers and the event’s interactive format was also agreed upon (eg a balance was agreed on the level of interaction versus the number and range of speakers at the event) based on listening to each other’s perspectives. To reach these agreements, SK and AT communicated via regular emails and feedback to APAB members (during monthly meetings) and the research team (during bi-weekly in-person progress meetings) respectively. SK and AT also held separate in-person meetings a minimum of once a month and corresponded regularly by email and phone.

**Figure 2:**
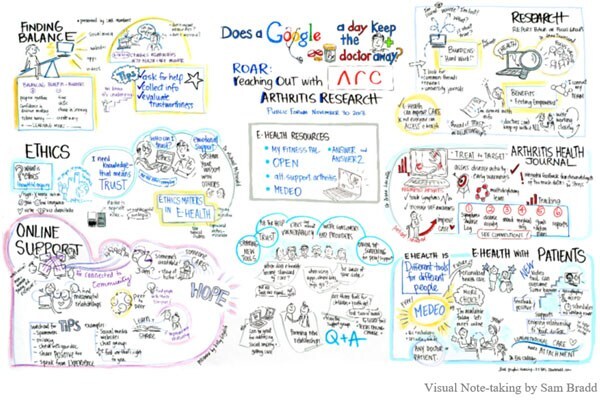
Visual note-taking of the session

APAB members contributed organizational, leadership, communication and other skills and resources to the planning process, which were relied upon by the researchers. For example, a committee of five APAB members led by SK set key milestones and oversaw progress to achieving them. The committee independently secured the event venue, and requested EC (employed by APAB as a Research Liaison with funding allocated by Arthritis Research Canada) to arrange webcasting services for the event. One APAB member with an employment background in marketing endorsed a graphic illustrator (proposed by AT and JL) to do live visual note-taking of the session (see Figure 2).

Another APAB member (who was also a physiotherapist) prepared stretch breaks, and another member (who was also a professional actor) worked with AT on the analysis of research data to present findings as a role-play [23]. Also led by SK, a separate committee of two APAB members developed the event’s budget, including allocating funds from APAB’s budget, and a smaller amount from the research grant co-led by AT and CB [21,22]. Furthermore, SK recruited APAB members to provide feedback on presenters’ slides for clarity and lay language in advance, organize catering, set up the venue and audio-visual equipment, or present or co-present on the day of the event.

Collaborators also held a teleconference to invite AM, a patient advocate/activist living with arthritis to moderate online conversations about the event, on account of her expertise in engaging with healthcare stakeholders using social media and her existing online network. Based in Toronto, AM advised on how to create an appropriate hashtag, generate interest on Twitter in advance of the event to maximize the number of online attendees on the day, and use social media analytics to assess the impact of the event. AM also prepared content to stimulate social media discussion about the event in advance and during the presentations. Thus, patient leaders drove the planning process of the event, anticipating how to engage with audiences, and contributing a range of valuable resources, skills and expertise, which researchers may not otherwise have had access to. These contributions were sincerely valued within the patient-researcher collaboration, which strengthened mutual respect for each other’s roles and priorities throughout the planning and preparation phase.

## The Event

**Figure 3:**
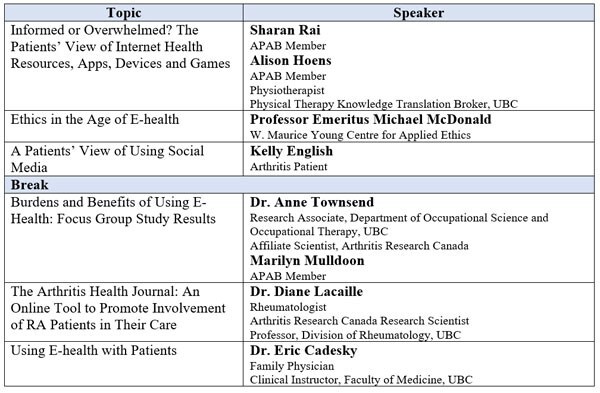
Topics and speakers for eROAR2013

APAB members and researchers worked together during the event to stimulate interaction between local, national and international stakeholders via multiple methods of engagement. While both patient and researcher collaborators were on-hand to greet the 52 in-person audience members, EC moderated a live webcast that reached 117 total views (7% from outside Canada) and, as the online facilitator, AM stimulated a conversation on Twitter involving 42 participants (62% from outside Canada). APAB collaborators also supported CB whose role it was to include online participants in the in-person conversation. Of the six presentations, three were given by APAB members and a patient (who subsequently joined APAB) with expertise in social media, who shared their first-hand knowledge on Internet health resources, apps, devices and games, and online support groups (Figure 3) [24,25].

In turn, the patient priorities were emphasised as potential areas of future research. An ethicist, a clinician scientist, and a family physician also gave presentations and discussed research relevant to the theme of the event, positioned as experts alongside the patient expertise (Figure 3) [23,26,27]. In these ways, the choice of speakers and presentation topics illustrated a break with the traditional hierarchy of scientific knowledge and patient or lay experience. APAB and researchers were able to work together to develop an inclusive, accessible and engaging event in which different perspectives and various forms of knowledge (eg experiential, scientific) were mutually welcomed and exchanged.

## Challenges, Reflections, Solutions

One challenge for patients and researchers was negotiating the patient-led aspect of eROAR2013 and the associated effort this meant (while acknowledging potential burdens for patients). Given the wide range of experience and skills of the APAB members, those less-experienced felt uncertainty about the tasks they undertook and sought guidance and support from other more experienced patient collaborators and researchers. A respectful approach taken by patient and researcher collaborators recognized this diversity within APAB members, in their specialist skills, knowledge and varying degrees of experiences, as well as different life situations and stages of illness, which impacted the nature and level of their engagement. In addition to balancing expectations of APAB members’ roles and responsibilities in the context of daily lives, it would be helpful for more experienced patient and researcher collaborators to provide more induction, mentorship and training to less experienced APAB members.

Challenges also emerged in the collaborative decision making process, for example in the event planning. In order to reach an agreed balance between academic terminology and every-day language to promote the event, the collaborators spent significant time in discussion, working together in a joint intellectual effort [28]. This process meant delays to the scheduled release of promotional materials, and contributed additional unanticipated hours that had not been bracketed into already busy schedules (eg involving work, managing health, travel and other daily life contingencies). Also, both APAB and researcher collaborators found it challenging to clarify the expectations and responsibilities of the remote patient’s role because it was unprecedented at a ROAR event. To prepare for moderating the conversation on Twitter, AM independently sourced a significant amount of information. It was particularly difficult to predict in advance how much time would be required to perform this role, and to plan ongoing support effectively. One potential solution could be for collaborators to develop a guide to simplify the steps involved in hosting a chat on Twitter in advance, covering details such as registration of the hashtag (#eROAR13), publicity, and receiving presenter slides in a timely manner.

APAB collaborators reflected that they valued learning about the latest arthritis research during discussions, while researchers valued the training they received from APAB collaborators on how to better engage lay audiences with their research, the specialist skills and expertise they provided and the insights into their experience of collaborating in the context of their daily lives. This recognition and appreciation for mutual learning and respect built on the established research partnership, and made reflecting on the challenges more comfortable. In this way, trust and respect underpinned collaborative decision making that recognised differences in expertise, skills, experiences and priorities. By perceiving patient collaborators as experts in their own right, rather than experts in the researchers’ own image (whereby training may be needed for patients to conduct research), the more traditional hierarchy of knowledge was dampened. In its place was a mutual appreciation of the diverse skills that drove the collaboration, which in this instance was a patient led KT event that encapsulated the concept of “Nothing About Us Without Us” in principle and in practice.

The case study we report offers an opportunity to expand on the fledgling practice of patient engagement often reported in existing literature, such as that of INVOLVE and Hewlett [15].

It was a cooperative experience that can contribute to refining our thinking and enactment of patient engagement as it develops in the context of established patient-researcher partnerships [16].

## Conclusions

Building successful, effective and meaningful patient engagement in research is a multi-layered, sometimes challenging, and valuable process that continues to evolve. In a knowledge translation event held in Vancouver, Canada, strong relationships built over time laid the foundation for a patient-led collaboration that revealed a different type of patient engagement than is typically reported. In describing the responsibilities and practical tasks undertaken, values and ethical considerations (eg, mutuality, understanding, respect and diversity) that underpin patient engagement in research are revealed as they are enacted relationally in a participatory space. It is our hope that this paper will help others to reflect on the changing nature of patient-researcher collaboration. We welcome feedback on our description and reflections on this case study.

## Footnotes

Endnotes a Formerly known as the Consumer Advisory Board, the Arthritis Patient Advisory Board chose to change its name in 2014 as it was felt the term “consumer” denoted an individual who purchased information or health care by choice. Members felt more comfortable identifying with the term “arthritis patient,” as it was considered to be more accurate while no longer seeming to indicate passivity as it had done when the original name was chosen.

b At the time of writing, APAB includes 23 members and 12 alumni. (29 women; 6 men).

c Figure 1 includes data up to and including the eROAR13 event. Further information about ROAR events (including those after 2013) is archived on Arthritis Research Canada’s website at www.arthritisresearch.ca.
